# Quantitative multichannel NC-AFM data analysis of graphene growth on SiC(0001)

**DOI:** 10.3762/bjnano.3.19

**Published:** 2012-02-29

**Authors:** Christian Held, Thomas Seyller, Roland Bennewitz

**Affiliations:** 1 INM – Leibniz-Institute for New Materials, Campus D2 2, 66123 Saarbrücken, Germany; 2 Lehrstuhl für Technische Physik, Universität Erlangen-Nürnberg, 91058 Erlangen, Germany

**Keywords:** FM-AFM, graphene, 6H-SiC(0001), KPFM, SPM

## Abstract

Noncontact atomic force microscopy provides access to several complementary signals, such as topography, damping, and contact potential. The traditional presentation of such data sets in adjacent figures or in colour-coded pseudo-three-dimensional plots gives only a qualitative impression. We introduce two-dimensional histograms for the representation of multichannel NC-AFM data sets in a quantitative fashion. Presentation and analysis are exemplified for topography and contact-potential data for graphene grown epitaxially on 6H-SiC(0001), as recorded by Kelvin probe force microscopy in ultrahigh vacuum. Sample preparations by thermal decomposition in ultrahigh vacuum and in an argon atmosphere are compared and the respective growth mechanisms discussed.

## Introduction

Graphene grows epitaxially on the Si face of 6H-SiC(0001) by thermal decomposition in vacuum or an inert atmosphere. Recently, fundamental studies have led to an improvement of this process, now allowing for the production of almost wafer-size single-layer graphene coverage [1–3]. Understanding the interaction between the substrate and the epitaxial layer during the growth process is crucial for further optimization. Towards this goal, the graphene layer thickness has been determined by various methods including scanning tunnelling microscopy (STM) [4], Raman spectroscopy [5], low-energy electron microscopy [6–7], transmission electron microscopy [8], and atomic force microscopy (AFM) [9–10]. AFM also allows the identification of the graphene layer thickness from the local contact potential as determined by means of Kelvin probe force microscopy (KPFM) [11–12]. As a further advantage, KPFM determines step heights more accurately than STM or AFM with constant bias [13] and is therefore employed in this study to investigate the growth mechanisms of graphene on SiC(0001).

The carbon for graphene growth on SiC(0001) is obtained from thermal decomposition of the bulk substrate. Heating the sample to temperatures above 1100 °C leads to Si evaporation and to the formation of carbon-rich reconstructions [3]. At even higher temperatures these processes lead to the growth of graphene. A high homogeneity of the graphene coverage was obtained in ultrahigh vacuum by cyclic heating to 1200 °C [2] and in an argon atmosphere by prolonged heating to 1650 °C [1]. On the Si face of the 6H-SiC(0001) wafers the thickness of the graphene layer is limited to two or three layers. The layer coverage is controlled by the growth temperature rather than by the duration of the heating cycle [14]. The determination of substrate step heights and of related changes in the graphene coverage has already provided interesting insight into the possible growth mechanisms. For example, Charrier et al. observed a preferred step height of one half of a unit cell of 6H-SiC(0001) after thermal decomposition [15]. Lauffer et al. correlated these steps with a change in the graphene coverage, based on the observation that one half of a unit cell has almost the same carbon density as one layer of graphene [16].

## Experimental

Noncontact atomic force microscopy (NC-AFM) measurements were performed in ultrahigh vacuum (UHV, *p* < 2·10^−10^ mbar) by means of a home-built microscope similar to the one described in [17]. Kelvin probe force microscopy (KPFM) studies were performed in the frequency-modulation mode [18–19]. The modulation frequency was set to 1000 Hz with a bias amplitude of 200 mV. Polycrystalline diamond-coated tips (nanosensors) with a typical radius of 20 to 70 nm were used. Frequencies for the first normal mode of the cantilever were around 100 kHz. This choice of cantilever gives the opportunity to perform complementary contact-mode friction and noncontact KPFM experiments on the same surface areas [20].

### Graphene grown in UHV

**Figure 1:**
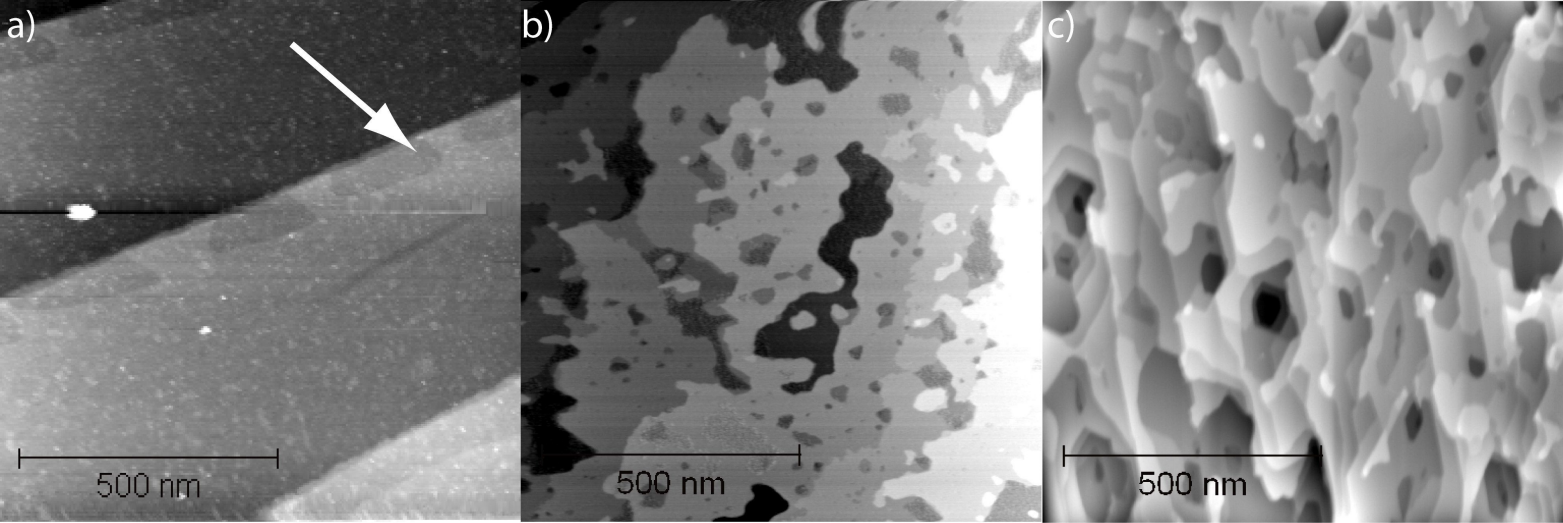
Topography images of the SiC(0001) sample (a) before annealing, (b) after oxide removal at 1000 °C, and (c) after graphene growth at 1300 °C. Step heights in (a) are 1.5 nm between the large terraces and 0.25 nm towards the small depressed islands (indicated by the arrow). Step heights in (b) are 0.25, 0.50 and 0.75 nm, evenly distributed. Step heights in (c) vary from 0.09 nm to 0.75 nm.

The substrate material for the study is the Si face of 6H-SiC(0001). The unit cell of 6H-SiC is composed of six bilayers of SiC(0001) each with a height of 0.25 nm. Wafers of 6H-SiC(0001) were purchased from SiCrystal AG. Polishing scratches were removed by hydrogen etching (grade 5.0, *p* = 1 bar, *T* = 1550 °C, *t* = 15 min) [1]. After insertion into UHV and heating to 120 °C for 10 h to remove adsorbed water, the surface was imaged by NC-AFM (Figure 1a). Flat terraces with a typical width of 500 nm were found. The surface of terraces is covered with irregular mounds of up to 0.5 nm in height. Smaller depressed islands decorate the steps between terraces (see white arrow in Figure 1a). The steps between terraces have a typical height of 1.5 nm, whereas the smaller steps towards the depressed islands have a height of roughly 0.25 nm (Table 1). The step heights match the height of the SiC unit cell of 1.52 nm [21] and the SiC bilayer height of 1.52 nm/6 = 0.253 nm, respectively.

**Table 1:** Table of different step heights found before and after graphenization of 6H-SiC(1000). Dominant step heights are underlined. After graphenization the substrate step heights formed as multiples of the SiC(0001) bilayer height of 0.25 nm may vary by the graphene thickness of 0.33 nm.

Substrate	Step heights found	Figure

Wafer SiC as received	1.5 and 0.25 nm	Figure 1a
Graphenized in argon	0, 0.25, 0.5, 0.75 , 1, …, 2 nm ± 0.33 nm for each of the above.	Figure 2a, Figure 3a, Figure 4b
Wafer SiC heated to 1000 °C	0.25, 0.50, 0.75 nm	Figure 1b
Graphenized in UHV	0.75 nm ± 0.33 nm	Figure 1c, Figure 2a, Figure 4a

**Figure 2:**
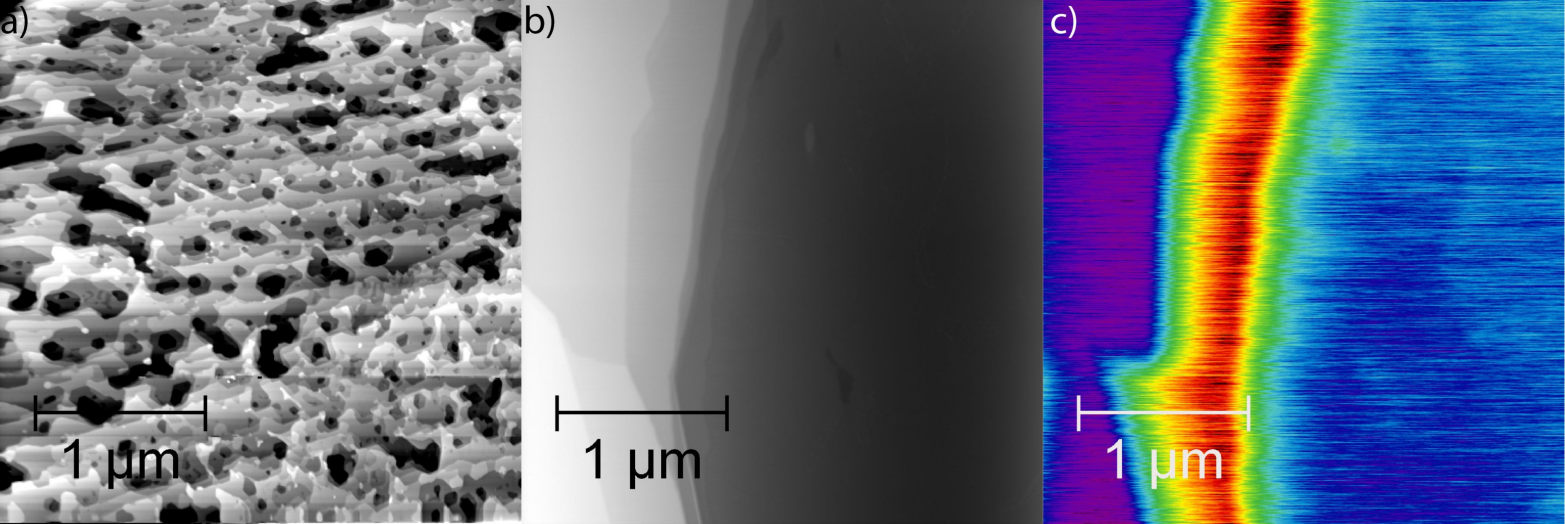
Topography images of graphene layers epitaxially grown on SiC(0001); (a) preparation in UHV, (b) preparation in an argon atmosphere. Step heights in (a) vary from 0.09 nm up to 0.75 nm. The total height of the step bunch in (b) is 3.25 nm. The contrast in the contact potential in (c) was recorded simultaneously with the topography in (b). Blue areas indicate single-layer graphene; red areas with 130 mV higher contact potential indicate double-layer graphene.

The surface oxide was removed in UHV by direct-current heating (*T* = 1000 °C, *t* = 6 min) [3]. The temperature was determined with an infrared pyrometer adjusted to an emissivity of 0.9. This oxide removal technique is known to change the SiC surface stoichiometry, as the oxide layer is removed by evaporation of SiO gas. Overall, the surface structure remains the same upon oxide removal (Figure 2b). The width of the large terraces is slightly reduced and a number of smaller and larger pits and islands with lateral extensions of only a few nanometers up to hundreds of nanometers are found. Except for a few remaining rough spots the surface is now atomically smooth. Step heights between the smooth terraces are mostly 0.25 nm, 0.5 nm and 0.75 nm, which again correspond to multiples of the SiC(0001) bilayer height (Table 1). The step height between rough spots and adjacent smooth terraces was found to be approximately 0.17 nm in good agreement with previous studies[10].

Graphene was grown by first heating the sample to 1000 °C for 6 min to remove contaminants and also to reduce the pressure burst during the subsequent graphenization step of heating to 1300 °C for 30 s [3]. This treatment changes the topography significantly (Figure 1c). The largest atomically flat areas now have a lateral extension of only 100 nm. The sample is covered with small pits of hexagonal shape. A large variety of step heights is found (Table 1).

### Graphene grown in an argon atmosphere

The same starting material and sample preparation, i.e., wafer manufacturer, polishing, and hydrogen etching, were used for the graphenization in an argon atmosphere at 1650 °C following the procedure describe in [1]. After graphenization the sample was introduced into the UHV chamber and heated for 10 h at 120 °C in order to remove adsorbed water.

A direct comparison of samples prepared in UHV and in an argon atmosphere reveals huge differences in the surface topography (Figure 2a and Figure 2b). While the sample prepared in UHV exhibits the pitted structure described above, the sample prepared in an argon atmosphere shows only a few straight step bunches every several microns.

## Results

**Figure 3:**
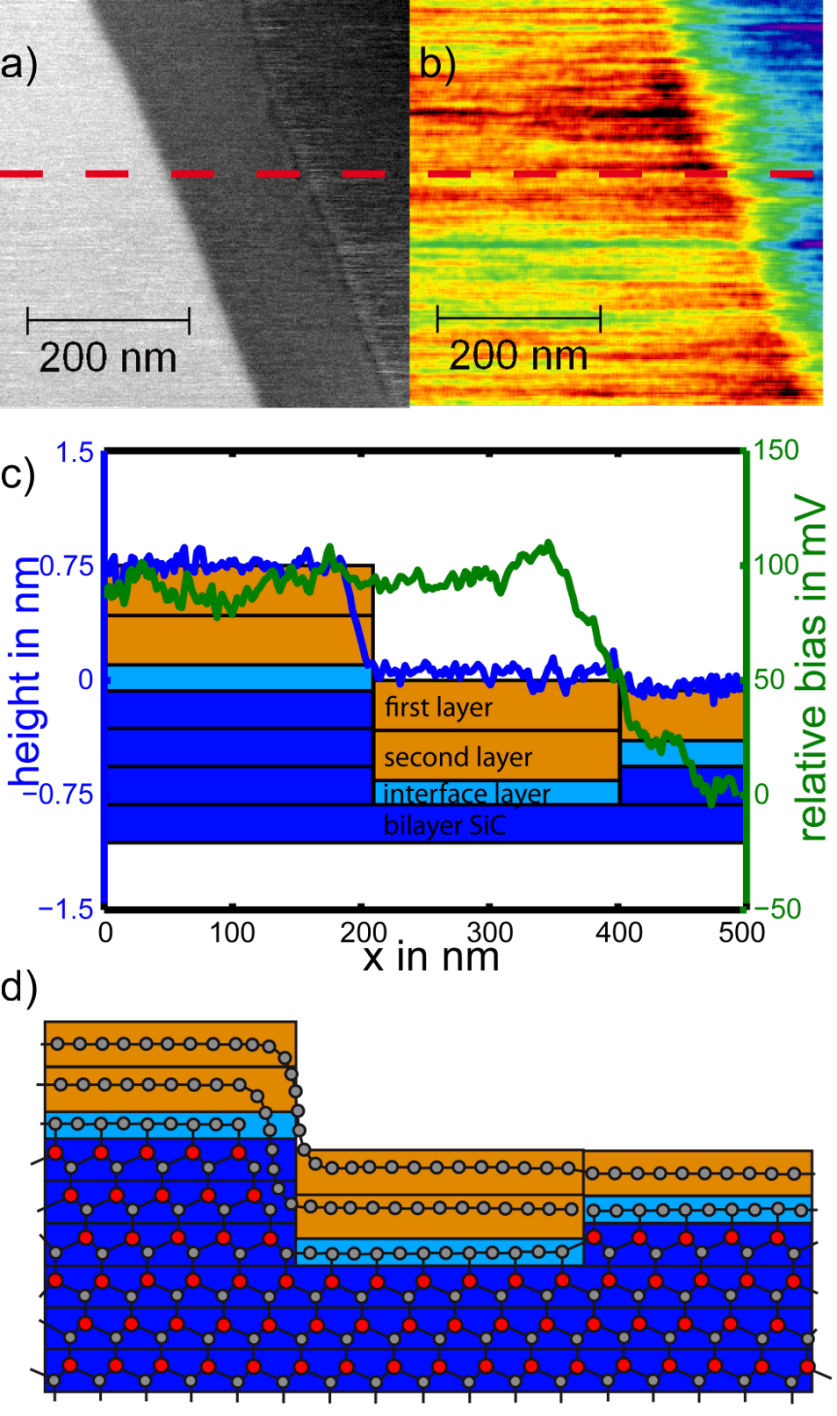
(a) Topographic image showing two steps found typically on samples prepared in an argon atmosphere. (b) Corresponding image of the contact potential difference. Note that only the small step in (a) coincides with a shift in contact potential. (c) Topography (blue) and contact-potential (green) profiles taken along the dashed line in (a). Underlying is a schematic illustration of the corresponding substrate composition. Different layers are drawn to their corresponding step height as SiC(0001) bilayer (0.25 nm, blue), interfacial layer (unknown height, light blue), and graphene layer (0.33 nm, orange). (d) Schematic atomic model of the surface structure, showing SiC bilayers, the carbon-rich interface layer, and single- and double-layer graphene.

KPFM measurements reveal variations in the graphene coverage as contributing to the different step heights observed. Figure 3 shows a typical step structure for a sample prepared in an argon atmosphere. Of the two topographic steps (Figure 3a) only one coincides with a change in contact potential (Figure 3b). The underlying surface structure is analyzed in Figure 3c and represented in an atomic ball-and-stick scheme in Figure 3d. The left step is a substrate step of three bilayers of SiC with a height of 0.75 nm, indicated by the three blue blocks representing the bilayers. The right step is a substrate bilayer step combined with a change in graphene coverage from single to double layer. The resulting topographic step height is 0.09 nm, the change in contact potential 130 mV. Such analysis is supported by the fact that steps with a height that is a multiple of the SiC bilayer height never coincide with a change in contact potential. The interface layer introduced in Figure 3c has been reported as a graphitic layer covalently bound to the SiC substrate [4,16]. While its influence on the electronic structure and contact potential is under discussion, it has no influence on the step heights between graphene-covered terraces.

**Figure 4:**
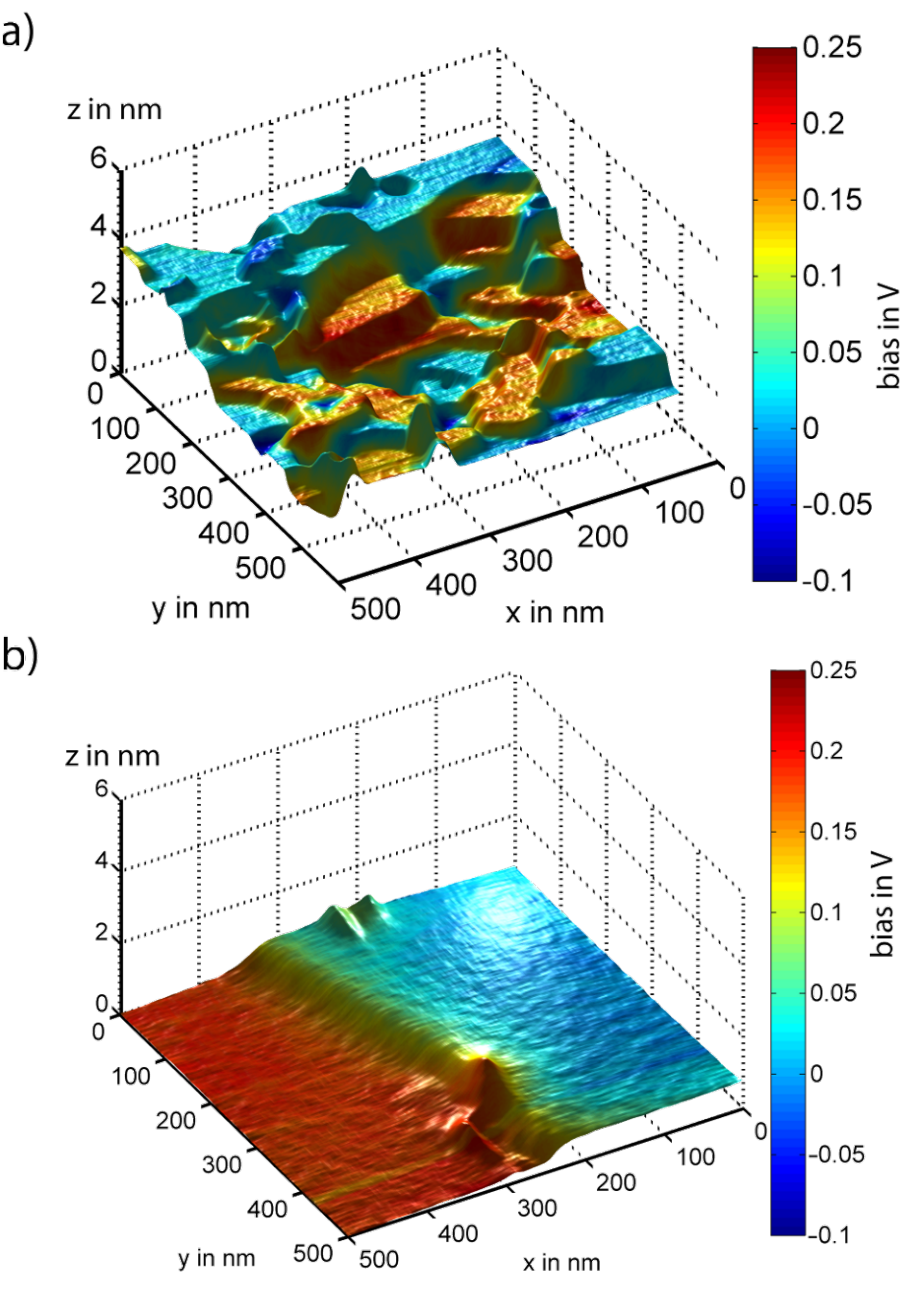
Rendered images of graphene layers on SiC(0001) prepared in (a) UHV and (b) an argon atmosphere. The colour represents the local contact potential. Bluish colour indicates single-layer graphene, reddish colour double-layer graphene.

Rendering the data sets into a pseudo-three-dimensional representation provides an intuitive understanding of the structure and composition of the sample [22]. Figure 4a shows results for a sample prepared in UHV. The topography data is rendered and overlayed with a colour scale representing the local contact potential. Most parts of the sample show a bluish colour indicating single-layer graphene coverage. Some smaller terraces exhibit a higher contact potential represented in red, which indicates double-layer graphene. Double-layer graphene spots are regularly observed to grow over a SiC bilayer substrate step. No change in contact potential is observed without a corresponding change in step height. The much simpler surface structure of samples prepared in an argon atmosphere is demonstrated in Figure 4b. The identification of surface areas such as the one in Figure 4b by KPFM allows subsequent experiments to be aimed at a direct comparison between single and double layer graphene, for example, in friction experiments.

While this visualization method allows for a quick identification of the surface structure, we will now introduce two-dimensional histograms as a complementary data representation. These histograms are very useful for a quantitative analysis of the complex structures of samples prepared in UHV.

**Figure 5:**
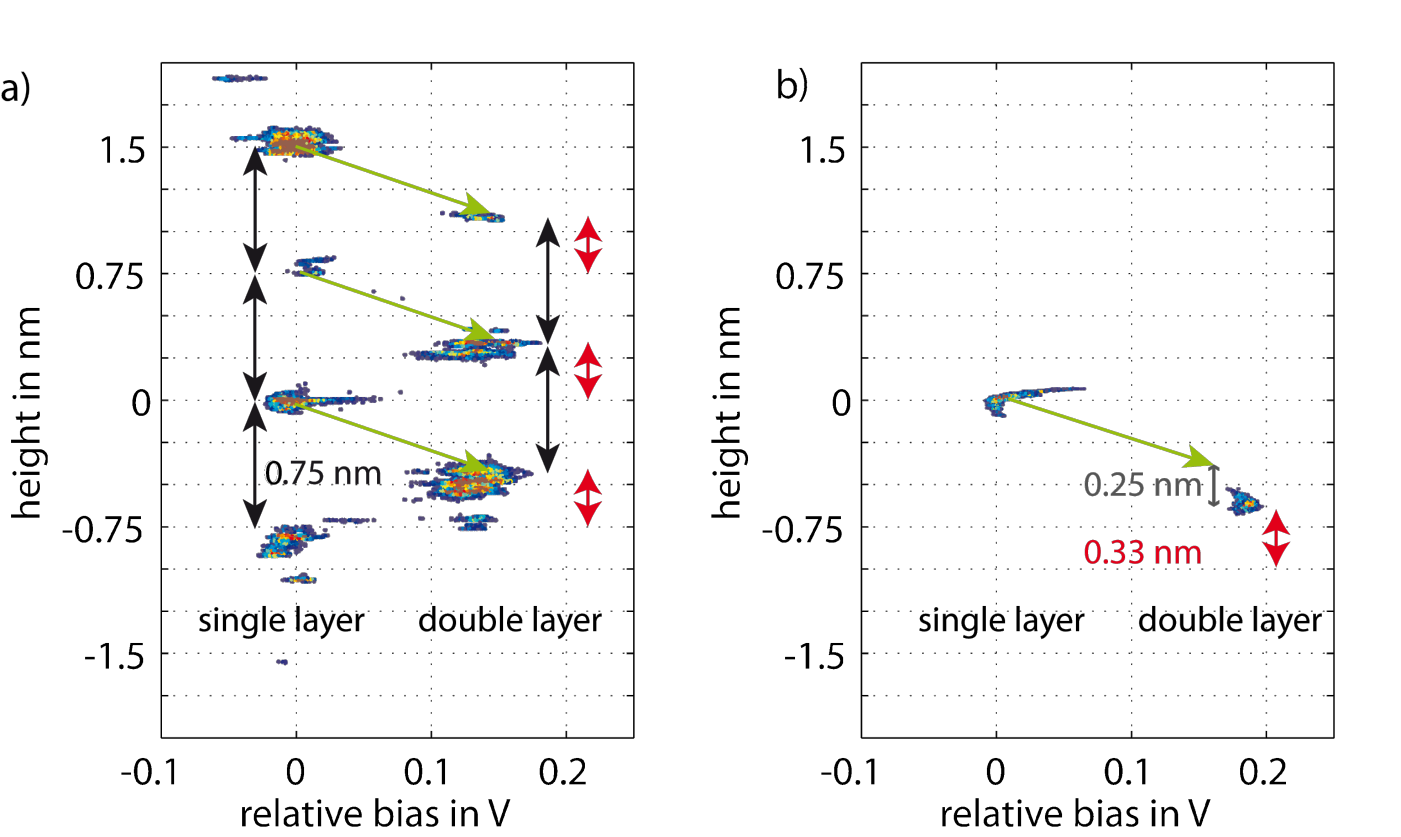
Two-dimensional histograms based on the data set for the rendered images in Figure 4. The colour scheme represents the number of data couples that fall into the respective topography and contact-potential bin; (a) sample prepared in UHV, (b) in an argon atmosphere. Black arrows indicate a height difference of 0.75 nm, equal to half a unit cell of 6H-SiC(0001), and the grey arrow a height of 0.25 nm, equal to one bilayer of SiC(0001). Red arrows indicate the step height of 0.33 nm corresponding to one graphene layer. Green arrows indicate a suggested graphene growth process, in which three SiC bilayers are consumed to produce one single graphene layer. Points with less than 5 counts are left transparent to enhance readability of the graph. The colour scale ranges from 5 (blue) to 70 (red) occurrences per 0.01 nm and 1.75 mV.

Histograms represent the distribution of values in a given data set. Here we are using two-dimensional histograms to represent the data contained in multichannel NC-AFM frames. Several signal values are assigned to each pixel of a scanned frame, e.g., topography and contact-potential values. Using topography and contact potential as axes of a two-dimensional scatter plot, the frequency of occurrence of each pair of topography and contact-potential values is represented by a colour scheme. In this way, topography and contact potential can be graphically correlated while their quantitative values can be directly read from the plot. In order to make two such histograms comparable, topography and contact-potential values are given with respect to the values found in one reference area of the scan frame. A scan frame recorded with 512 lines of 512 pixels provides 262144 data points for this scatter plot, enough for a distinct representation of the relationship between topography and contact potential. Figure 5 shows two-dimensional histograms based on the data sets already presented in the rendered images in Figure 4.

The sample prepared in UHV is analyzed in Figure 5a. Two distinct groups of clustered data points are lined up vertically, reflecting the coverage by single and double-layer graphene. Within each group, a distinct step height of 0.75 nm is dominant, which corresponds to half the unit cell of 6H-SiC(0001). The step height between single- and double-layer graphene terraces is typically 0.42 nm, indicated by green arrows in Figure 5a. It has been suggested that half a unit cell of SiC(0001) is consumed for the growth of one layer of graphene. This relation suggests itself as the density of carbon atoms is very similar for one half of a unit cell of SiC and one layer of graphene. The step height of 0.42 nm is then given as the difference between 0.75 nm for half a unit cell and 0.33 nm for the height of one layer of graphene.

The sample prepared in an argon atmosphere is analyzed in Figure 5b, its structure with wide terraces and few steps is reflected in the observation of only two narrow clusters of data points in the histogram. The two groups correspond to a height difference of 0.64 nm, i.e., about 0.33 nm less than four SiC(0001) bilayers, which is again the step height of the graphene layer. Therefore we conclude that the lower terrace is depressed by four SiC bilayers but is covered by one additional graphene layer compared to the upper terrace. The extra SiC bilayer decomposed for the structure in Figure 5b as compared to Figure 5a is indicated by the grey arrow.

## Discussion

The results described above shed light on the growth mechanism of graphene on the Si face of 6H-SiC(0001). After oxide removal at 1000 °C in UHV, the step heights vary between one, two and three bilayers of the SiC(0001) structure. Subsequent graphenization at 1300 °C in UHV results in a preferred step height of three bilayers of SiC(0001). Two mechanisms leading to this step height have been suggested. As discussed above, a little more than three bilayers SiC(0001) provide the carbon atoms required to form one graphene sheet [3]. This simple stochiometric argument is supported by our experimental results, as all spots for single-layer graphene coverage are connected to double-layer graphene spots by the corresponding green arrows in Figure 5. The contact potential difference between single- and double-layer graphene is always found to be close to 130 mV.

However, the stochiometric argument does not explain the preferred step height of 0.75 nm between single-layer graphene areas or between double-layer graphene areas. Hupalo et al. [2] have concluded that different SiC bilayers within the SiC(0001) unit cell have different Si evaporation rates, i.e., the first bilayer of each half unit cell evaporates fastest, followed by the second bilayer, whereas every third bilayer exhibits a low evaporation rate. In Figure 5a all height differences fit multiples of three SiC bilayers. Double layer graphene areas are shifted in height by exactly 0.33 nm, i.e., the thickness of one graphene layer. Therefore, single and double layers of graphene have grown on terraces defined by half unit cells of the 6H-SiC(0001) structure. Terraces not following this rule were found rarely, supporting the suggestion of Hupalo et al. for a mechanism of graphene growth in UHV.

Samples prepared in an argon atmosphere differ significantly in step structure. Atomically flat terraces extend over several microns. They are separated by bunches of steps reaching heights of up to 10 nm. The steps have heights that correspond to multiples of a bilayer of SiC(0001), varying from single up to seven SiC bilayers.

These results indicate that the mechanism described for growth in UHV is not the dominant mechanism for the step structure formation upon growth in argon. The terraces found after graphenization in argon are larger than those found on the starting material, excluding a simple carbon-maintaining transformation of the sample. Furthermore, step heights between the large terraces do not match the height of the half unit cell. The differences may be explained by enhanced diffusion at the elevated temperature of 1650 °C used for the preparation in argon as compared to 1300 °C for the preparation in UHV. Several studies have shown that the diffusion of carbon and silicon atoms differs significantly for the two temperatures, for which absolute values are still under discussion [23–25]. Diffusion of carbon atoms from areas with carbon excess to carbon-depleted areas appears to be a reasonable mechanism for the formation of larger terraces. Future models of the effect of diffusion will have to take into account the preferred nucleation of double-layer graphene at step bunches.

Samples prepared in argon show an interesting deviation of the contact potential difference between single and double layer graphene from the average value of 130 mV. Terraces that are separated by steps with a height other than a half unit cell of SiC(0001) exhibit contact potential differences of 130 ± 50 mV, examples are presented in Figure 2c and Figure 5b. We found no predictable relation between step height and contact potential difference in the available data. The origin of these deviations is not clear at present, but differences in the interface layer between graphene and SiC at different stacking positions within the unit cell are plausible candidates to explain the variations in contact potential. These differences could express themselves as a variation of the surface reconstruction (e.g., (5×5), (6×6) versus (6√3×6√3)R30°) of the interface layer [4].

Finally, we add a few comments on the data quality in the two-dimensional histograms. The contact potential signal recorded in KPFM shows only a little noise and drift, and can be directly processed in the form of histograms. In contrast, the topography signal needs to be processed to correct for the effects of drift, piezo creep, and piezo hysteresis [26]. The goal, and the justification, for processing is to obtain a minimal curvature of atomically flat terraces. Most NC-AFM operating in UHV do not offer the opportunity to linearize the piezo actuators in a closed-loop scheme. However, for ambient conditions such linearized instruments are commercially available and provide suitable input data for two-dimensional histograms, in particular when the lift-mode KPFM is used [27].
